# 10Kin1day: A Bottom-Up Neuroimaging Initiative

**DOI:** 10.3389/fneur.2019.00425

**Published:** 2019-05-09

**Authors:** Martijn P. van den Heuvel, Lianne H. Scholtens, Hannelore K. van der Burgh, Federica Agosta, Clara Alloza, Celso Arango, Bonnie Auyeung, Simon Baron-Cohen, Silvia Basaia, Manon J. N. L. Benders, Frauke Beyer, Linda Booij, Kees P. J. Braun, Geraldo Busatto Filho, Wiepke Cahn, Dara M. Cannon, Tiffany M. Chaim-Avancini, Sandra S. M. Chan, Eric Y. H. Chen, Benedicto Crespo-Facorro, Eveline A. Crone, Udo Dannlowski, Sonja M. C. de Zwarte, Bruno Dietsche, Gary Donohoe, Stefan Du Plessis, Sarah Durston, Covadonga M. Díaz-Caneja, Ana M. Díaz-Zuluaga, Robin Emsley, Massimo Filippi, Thomas Frodl, Martin Gorges, Beata Graff, Dominik Grotegerd, Dariusz Gąsecki, Julie M. Hall, Laurena Holleran, Rosemary Holt, Helene J. Hopman, Andreas Jansen, Joost Janssen, Krzysztof Jodzio, Lutz Jäncke, Vasiliy G. Kaleda, Jan Kassubek, Shahrzad Kharabian Masouleh, Tilo Kircher, Martijn G. J. C. Koevoets, Vladimir S. Kostic, Axel Krug, Stephen M. Lawrie, Irina S. Lebedeva, Edwin H. M. Lee, Tristram A. Lett, Simon J. G. Lewis, Franziskus Liem, Michael V. Lombardo, Carlos Lopez-Jaramillo, Daniel S. Margulies, Sebastian Markett, Paulo Marques, Ignacio Martínez-Zalacaín, Colm McDonald, Andrew M. McIntosh, Genevieve McPhilemy, Susanne L. Meinert, José M. Menchón, Christian Montag, Pedro S. Moreira, Pedro Morgado, David O. Mothersill, Susan Mérillat, Hans-Peter Müller, Leila Nabulsi, Pablo Najt, Krzysztof Narkiewicz, Patrycja Naumczyk, Bob Oranje, Victor Ortiz-Garcia de la Foz, Jiska S. Peper, Julian A. Pineda, Paul E. Rasser, Ronny Redlich, Jonathan Repple, Martin Reuter, Pedro G. P. Rosa, Amber N. V. Ruigrok, Agnieszka Sabisz, Ulrich Schall, Soraya Seedat, Mauricio H. Serpa, Stavros Skouras, Carles Soriano-Mas, Nuno Sousa, Edyta Szurowska, Alexander S. Tomyshev, Diana Tordesillas-Gutierrez, Sofie L. Valk, Leonard H. van den Berg, Theo G. M. van Erp, Neeltje E. M. van Haren, Judith M. C. van Leeuwen, Arno Villringer, Christiaan H. Vinkers, Christian Vollmar, Lea Waller, Henrik Walter, Heather C. Whalley, Marta Witkowska, A. Veronica Witte, Marcus V. Zanetti, Rui Zhang, Siemon C. de Lange

**Affiliations:** ^1^Connectome Lab, CTG, CNCR, VU Amsterdam, Amsterdam, Netherlands; ^2^UMC Utrecht Brain Center, Department of Psychiatry, University Medical Center Utrecht, Utrecht, Netherlands; ^3^Department of Neurology, UMC Utrecht Brain Center, University Medical Center Utrecht, Utrecht, Netherlands; ^4^Neuroimaging Research Unit, Institute of Experimental Neurology, Division of Neuroscience, IRCCS San Raffaele Scientific Institute, Vita-Salute San Raffaele University, Milan, Italy; ^5^Division of Psychiatry, University of Edinburgh, Edinburgh, United Kingdom; ^6^Department of Child and Adolescent Psychiatry, IiSGM, CIBERSAM, School of Medicine, Hospital General Universitario Gregorio Marañón, Universidad Complutense, Madrid, Spain; ^7^Department of Psychiatry, Autism Research Centre, University of Cambridge, Cambridge, United Kingdom; ^8^Department of Neonatology, UMC Utrecht Brain Center, Wilhelmina Children's Hospital, University Medical Center Utrecht, Utrecht, Netherlands; ^9^Department of Neurology, CRC “Obesity Mechanisms”, Subproject A1, Max Planck Institute for Human Cognitive and Brain Sciences, University of Leipzig, Leipzig, Germany; ^10^Department of Psychology, Concordia University, Montreal, QC, Canada; ^11^Department of Child Neurology, UMC Utrecht Brain Center, Wilhelmina Children's Hospital, University Medical Center Utrecht, Utrecht, Netherlands; ^12^Laboratory of Psychiatric Neuroimaging (LIM21), Faculdade de Medicina, Instituto de Psiquiatria, Hospital das Clinicas HCFMUSP, Universidade de São Paulo, São Paulo, Brazil; ^13^Clinical Neuroimaging Laboratory, Centre for Neuroimaging and Cognitive Genomics (NICOG), NCBES Galway Neuroscience Centre, College of Medicine Nursing and Health Sciences, National University of Ireland Galway, Galway, Ireland; ^14^Department of Psychiatry, Faculty of Medicine, Chinese University of Hong Kong, Hong Kong, China; ^15^Department of Psychiatry, University of Hong Kong, Hong Kong, China; ^16^Psychiatry Unit, Department of Medicine and Psychiatry, Hospital Universitario Marques de Valdecilla, IDIVAL, CIBERSAM, Hosptial Universitario Virgen del Rocío, Universidad de Seville, Seville, Spain; ^17^Brain and Development Research Center, Leiden University, Leiden, Netherlands; ^18^Department of Psychiatry, University of Münster, Münster, Germany; ^19^Department of Psychiatry, University of Marburg, Marburg, Germany; ^20^Cognitive Genetics and Cognitive Therapy Group, Neuroimaging and Cognitive Genomics Centre and NCBES Galway Neuroscience Centre, School of Psychology and Discipline of Biochemistry, National University of Ireland, Galway, Ireland; ^21^Department of Psychiatry, Stellenbosch University, Cape Town, South Africa; ^22^Research Group in Psychiatry GIPSI, Department of Psychiatry, Faculty of Medicine, Universidad de Antioquia, Medellín, Colombia; ^23^Department of Psychiatry and Psychotherapy, University Hospital, Otto von Guericke University, Magdeburg, Germany; ^24^Department of Neurology, University of Ulm, Ulm, Germany; ^25^Department of Hypertension and Diabetology, Medical University of Gdańsk, Gdańsk, Poland; ^26^Department of Neurology of Adults, Medical University of Gdańsk, Gdańsk, Poland; ^27^Parkinson's Disease Research Clinic, Brain and Mind Centre, University of Sydney, Sydney, NSW, Australia; ^28^Department of Medicine and Therapeutics, Chinese University of Hong Kong, Hong Kong, China; ^29^Department of Psychiatry and Center for Mind, Brain and Behaviour, University of Marburg, Marburg, Germany; ^30^Institute of Psychology, University of Gdańsk, Gdańsk, Poland; ^31^Division of Neuropsychology, University of Zurich, Zurich, Switzerland; ^32^Department of Endogenous Mental Disorders, Mental Health Research Center, Moscow, Russia; ^33^Institute for Neuroscience and Medicine 7, Forschungszentrum Jülich, Jülich, Germany; ^34^Clinic of Neurology, School of Medicine, University of Belgrade, Belgrade, Serbia; ^35^Division of Psychiatry, University of Edinburgh, Edinburgh, United Kingdom; ^36^Laboratory of Neuroimaging and Multimodal Analysis, Mental Health Research Center, Moscow, Russia; ^37^Department of Psychiatry and Psychotherapy, Division of Mind and Brain Research, Charité - Universitätsmedizin Berlin, Berlin, Germany; ^38^University Research Priority Program “Dynamics of Healthy Aging”, University of Zurich, Zurich, Switzerland; ^39^Mood Disorders Program, Research Group in Psychiatry GIPSI, Department of Psychiatry, Faculty of Medicine, Hospital Universitario San Vicente Fundación, Universidad de Antioquia, Medellín, Colombia; ^40^Frontlab, Centre National de la Recherche Scientifique, Institut du Cerveau et de la Moelle Épinière, UMR 7225, Paris, France; ^41^Department of Psychology, Humboldt Universität zu Berlin, Berlin, Germany; ^42^School of Medicine, Life and Health Sciences Research Institute (ICVS), University of Minho, Braga, Portugal; ^43^Department of Psychiatry, Bellvitge Biomedical Research Institute-IDIBELL and CIBERSAM, Barcelona, Spain; ^44^Department of Molecular Psychology, Institute of Psychology and Education, Ulm University, Ulm, Germany; ^45^Psychiatry Unit, Department of Medicine and Psychiatry, IDIVAL, CIBERSAM, Hospital Universitario Marques de Valdecilla, Santander, Spain; ^46^Research Group, Instituto de Alta Tecnología Médica, Universidad de Antioquia, Medellín, Colombia; ^47^Priority Centre for Brain and Mental Health Research, The University of Newcastle, Newcastle, NSW, Australia; ^48^Laboratory of Psychiatric Neuroimaging (LIM21), Faculdade de Medicina, Instituto de Psiquiatria, Hospital das Clinicas HCFMUSP, Universidade de São Paulo, São Paulo, Brazil; ^49^2nd Department of Radiology, Medical University of Gdańsk, Gdańsk, Poland; ^50^Laboratory of Psychiatric Neuroimaging (LIM21), Departamento de Psiquiatria, Faculdade de Medicina, Universidade de São Paulo, São Paulo, Brazil; ^51^BarcelonaBeta Brain Research Center, Pasqual Maragall Foundation, Barcelona, Spain; ^52^Department of Psychiatry (IDIBELL and CIBERSAM) and Department of Psychobiology and Methodology in Health Sciences (UAB), Bellvitge Biomedical Research Institute-IDIBELL, CIBERSAM and Universitat Autònoma de Barcelona, Barcelona, Spain; ^53^Laboratory of Neuroimaging and Multimodal Analysis, Mental Health Research Center, Moscow, Russia; ^54^Neuroimaging Unit, Technological Facilities, Valdecilla Biomedical Research Institute IDIVAL, CIBERSAM, Santander, Spain; ^55^Institute for Neuroscience and Medicine 7/Institute of Systems Neuroscience, Forschungszentrum Jülich - Heinrich Heine Universitaet Duesseldorf, Jülich, Germany; ^56^Clinical Translational Neuroscience Laboratory, Department of Psychiatry and Human Behavior, University of California, Irvine, Irvine, CA, United States; ^57^Department of Child and Adolescent Psychiatry/Psychology, Erasmus Medical Center, Rotterdam, Netherlands; ^58^Donders Institute for Brain, Cognition, and Behaviour, Radboud University Medical Center, Nijmegen, Netherlands; ^59^Departments of Neurology, Cognitive Neurology, Max Planck Institute for Human Cognitive and Brain Sciences, University of Leipzig, Leipzig, Germany; ^60^Departments of Psychiatry, Anatomy and Neurosciences, Amsterdam UMC, Amsterdam, Netherlands; ^61^Department of Neurology, Epilepsy Centre, University of Munich Hospital, Munich, Germany; ^62^Division of Mind and Brain Research, Department of Psychiatry and Psychotherapy CCM, Charité Universitätsmedizin Berlin, Corporate Member of Berlin Institute of Health, Freie Universität Berlin, Humboldt-Universität zu Berlin, Berlin, Germany; ^63^Division of Mind and Brain Research, Department of Psychiatry and Psychotherapy CCM, Charité - Universitätsmedizin Berlin, Corporate Member of Berlin Institute of Health, Freie Universität Berlin, Humboldt-Universität zu Berlin, Berlin, Germany; ^64^Laboratory of Psychiatric Neuroimaging (LIM21), Faculdade de Medicina, Instituto de Psiquiatria, Hospital das Clinicas HCFMUSP, São Paulo, Brazil; ^65^Instituto de Ensino e Pesquisa, Hospital Sírio-Libanês, Universidade de São Paulo, São Paulo, Brazil; ^66^Department of Neurology, Max Planck Institute for Human Cognitive and Brain Sciences, Leipzig, Germany

**Keywords:** MRI, connectome analysis, diffusion weighted MRI, brain, network

## Abstract

We organized 10Kin1day, a pop-up scientific event with the goal to bring together neuroimaging groups from around the world to jointly analyze 10,000+ existing MRI connectivity datasets during a 3-day workshop. In this report, we describe the motivation and principles of 10Kin1day, together with a public release of 8,000+ MRI connectome maps of the human brain.

Ongoing grand-scale projects like the European Human Brain Project (1), the US Brain Initiative (2), the Human Connectome Project (3), the Chinese Brainnetome (4) and exciting world-wide neuroimaging collaborations such as ENIGMA (5) herald the new era of *big neuroscience*. In conjunction with these major undertakings, there is an emerging trend for bottom-up initiatives, starting with small-scale projects built upon existing collaborations and infrastructures. As described by Mainen et al. (6), these initiatives are centralized around self-organized groups of researchers working on the same challenges and sharing interests and specialized expertise. These projects could scale and open up to a larger audience and other disciplines over time, eventually lining up and merging their findings with other programs to make the bigger picture.

## 10Kin1day

One type of event that fits well with this grass-roots collaboration philosophy are short gatherings of scientists around a single theme, bringing together expertise and tools to jointly analyze existing neuroscience data. We organized 10Kin1day, an MRI connectome event, with the goal to bring together an international group of researchers in the field of neuroimaging and consistently analyze MRI connectivity data of the human cerebrum. We organized the event around five founding principles:

- use existing neuroimaging data, available from many research groups around the world; we focused on diffusion MRI data and aimed to bring together 10,000+ datasets
- analyze data from varying cohorts and imaging protocols, using a single, straightforward analysis strategy to encourage across-group collaborations and multisite studies
- perform all processing during a short workshop, with only basic expertise of analysis needed
- provide education on how to analyze resulting connectome data, so participants can continue to work on their projects after the event
- each participant analyzes their own data and is free to decide what to do with their analyzed results

## The 10K Workshop

**Figure 1:**
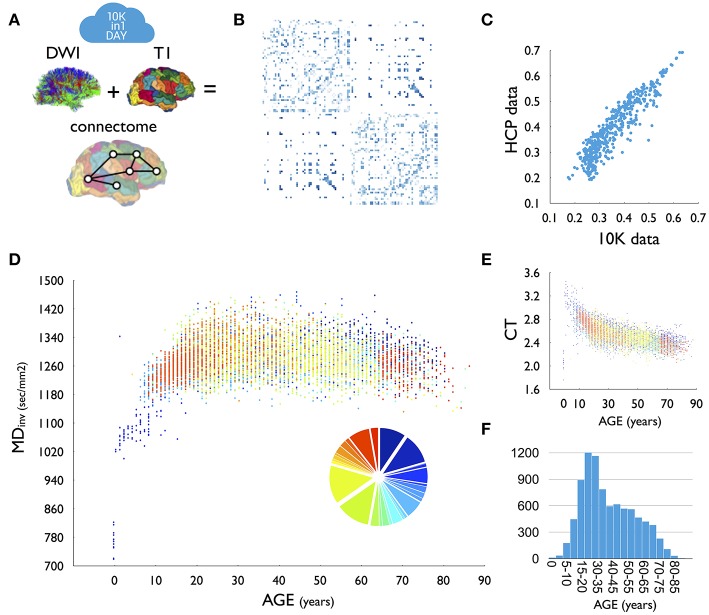
**(A)** For each dataset, DWI tractography was combined with T1-based parcellation of cerebral brain regions to reconstruct a brain network. **(B)** Group-averaged (group threshold 33%) FA matrix of the 10K dataset. **(C)** High overlap (*r* = 0.93) between group-averaged FA values as derived from high-resolution HCP data and the 10K dataset. **(D)** Relationship between age and average inverse mean diffusivity (MD, sec/mm2) across the 10K dataset. Colors indicate the different included datasets. Insert shows a pie diagram of the size of included datasets, color coded to set participation. One dataset (set_634413) was excluded from this plot, showing (across the age span) deviating FA (lower) and MD (higher) values than the other datasets (see methods). Due to the high total n, excluding this dataset did not change the relationship with age. **(E)** Relationship between age and average cortical thickness (CT). **(F)** Age distribution of the presented data as in **(D,E)**. T1, anatomical MRI; DWI, diffusion weighted imaging; CT, cortical thickness.

Over 50 participants from 40 different neuroimaging groups gathered in The Netherlands for a 3-day event. Participants brought and worked on their own datasets, varying from MRI data on healthy human brain organization, cross-sectional and longitudinal brain development, aging, cognitive psychology, as well as MRI data of a wide range of neurological and psychiatric brain disorders (including among others: Schizophrenia, Mood Disorders, Alzheimer's Disease, Mild Cognitive Impairment, Amyotrophic Lateral Sclerosis, Frontotemporal Dementia, Epilepsy and Parkinson's Disease). Written informed consent of the included healthy controls and/or patients was obtained by each of the participating researchers at their local institute. 10 TB online storage space and 50,000+ CPU hours was reserved on the Cartesius supercomputer of the collaborative Information and Communication Technology (ICT) organization for Dutch education and research (SURF, https://surfsara.nl/) to analyze the data during the workshop. Workshop participants performed data quality checks on their data 1 week before the event after which they uploaded the MRI data (Diffusion Weighted Images (DWI) and pre-processed T1 data, see Materials and Methods) to their own user account on the supercomputer. During the workshop, participants were brought up to speed on DWI processing, connectome construction (see section Materials and Methods for details on the performed analysis), and running parallel jobs on a supercomputer. Together, a total of 15,947 MRI datasets were processed into anatomical connectome maps, with each output dataset including connectivity matrices with different types of connection weights and multiple parcellation resolutions (Figures 1A,B). Data processing was paralleled by interactive educational talks and workshops on connectome analysis.

## Open Data

In line with the collaborative nature of the event, the 10K group discussed making the connectome maps available to the scientific community for non-commercial use, free of restrictions. We include herein the resulting individual connectome maps of 8,000+ connectome datasets across an age range of 0–90 years, with five different edge weights [number of traced streamlines (NOS), streamline density (SD), fiber length, fractional anisotropy (FA), and mean diffusivity (MD)] at three parcellation resolutions (80+ cortical and subcortical regions, 100+ and 200+ cortical regions, see section Materials and Methods for details). Connectome maps are presented anonymously and blinded for participation site, together with basic demographics (age in bins of 5 years, gender, patient/control status, Figure 1). Data is presented under the Non-Commercial Common Creative (CC BY-NC) license, free for all scientists to use in a non-commercial setting. A download request can be made at dutchconnectomelab.org for a download link to the data. Data for download includes connectivity matrices with five connectivity weights (NOS, FA, MD, fiber length, SD) at three atlas resolutions, information on the cortical and subcortical nodes, blinded group site and subject demographics (gender, age in 5 year bins, case/control).

## Concluding Words

We performed a few first analyses on the joint dataset, including cross-site consistency, comparison to Human Connectome Project (HCP) data and a first examination of effects of age (see Materials and Methods for more detail). We observed a high average consistency across sites with an average cross-site overlap of 92% (sd:0.0251) and a cross-site correlation of FA weights *r* = 0.88 (sd:0.0958), as well as a high consistency of the 10K group averaged matrix with data derived from the high-quality HCP, with at least 69% of pathways identified in HCP also observed in the 10K set and with 98% of all non-existing connections in HCP verified in the 10K set (Figure 1C). Furthermore, the distribution of weights across reconstructed connections is highly similar across the two datasets (FA weights, *r* = 0.93, *p* < 0.0001, Figure 1C). Age analysis shows clear developmental patterns of cortical morphology (Figure 1E) and white matter microstructure across age. Analysis of inverse MD showed rapid growth of microstructure in early years, with continuing development throughout adolescence, peaking around the beginning of the third decade, followed by a steady pattern of decline throughout aging (Figure 1D).

We acknowledge that there are many shortcomings to the presented MRI connectome dataset. Besides general, inherent limitations of diffusion MRI (7), the presented dataset is a collation of data from a wide variety of groups, acquired with different scanners, different scanning protocols, varying data quality etcetera, and includes data from a mixture of different patient and control populations. While these limitations place constraints on the type of investigations that one can perform with such collated multi-site datasets, we are optimistic that the 10K dataset can be used as a large reference dataset for future studies, enabling many technical and neuroscientific research questions to be addressed (e.g., Figure 1). As such, we hope that the presented data will be of use to the neuroscience community in the examination of the human connectome. Above all, we hope that our report will inspire others to organize exciting 10Kin1day-type of events in the near future, bringing together existing neuroimaging data and further catalyze open neuroimaging research of the healthy and diseased brain.

## Materials and Methods

A total of 42 groups (52 participants) participated in the workshop, some working on multiple datasets. Each dataset included a diffusion MRI scan and T1 MRI scan processed using FreeSurfer (8). Datasets across groups included data from 1.5 and 3 Tesla MRI with varying scanner protocols and number of applied DWI gradients. Data included MRI data of healthy participants and patients with a neurological or psychiatric disorder. Twenty-three groups were able to make their data available, making a total of 8,000+ connectome maps publicly available through means of this report. Reconstructed connectome maps are presented anonymously, coded for participation site and disease condition(s). Basic demographics of the datasets are included in the download set.

### DWI Preprocessing

DWI datasets were corrected for susceptibility and eddy current distortions using the open tools from the FMRIB Software Library (FSL, http://fsl.fmrib.ox.ac.uk). Depending on their DWI dataset, participants preprocessed their data using the FSL *eddy_correct* or *eddy* tool. For those DWI sets that included a subset of scans with an opposite k-space read out, an additional field distortion map was formed and applied to the DWI images (9).

### Cortical Parcellation

Before the event, the participants created FreeSurfer files based on their T1 images, with this output being subjected to varying degrees of quality control. The resulting parcellations of the cerebrum were used to select the regions of interest for the connectome reconstruction. The 68 cortical regions of FreeSurfer's standard Desikan-Killiany Atlas (10, 11) as well as 14 subcortical regions were selected as network regions. Additionally, FreeSurfer files were used to further parcellate the cortex into 114 and 219 regions, respectively using the Cammoun atlas (12).

### Fiber Reconstruction

After preprocessing of the DWI data, in-house developed scripts were used to fit a diffusion tensor to the diffusion signal in each voxel of the white matter mask (selected based on the white matter segmentation map of the FreeSurfer files) using robust tensor fitting (13). Simple Diffusion Tensor Imaging (DTI) reconstruction was used due to its robustness and relatively low sensitivity to false positive reconstructions compared to more advanced reconstruction methods (14), and thus potentially being the least distorting solution for connectome reconstruction and analysis based on MR imaging data (15). Decomposition of the tensor into eigenvectors and eigenvalues was used to select the main diffusion direction in each voxel, and to compute fractional anisotropy (FA) and mean diffusivity (MD) (16). Deterministic fiber tractography was used to construct large-scale white matter pathways. Eight seeds (evenly distributed across the voxel) started in each white matter voxel, and fiber streamlines were formed by following the main diffusion direction from voxel to voxel using the *fiber assignment by continuous tracking* (FACT) algorithm (17), until one of the stopping criteria was met. A streamline was stopped when (1) it hit a voxel with an FA < 0.1, (2) went out of the brain mask, or (3) made a turn >45 degrees.

### Connectome Reconstruction

A connectome map was made by combining the (sub)cortical parcellation map and the set of reconstructed fibers using commonly described procedures [see (18–21)]. For each of the Cammoun Desikan-Killiany parcellation maps (i.e., 14+68, 14+114, and 14+219 regions, respectively), the total collection of reconstructed fiber streamlines was used to assess the level of connectivity between each pair of (sub)cortical regions, represented as the *connectivity matrix CIJ*. (Sub)cortical regions were selected as the nodes of the reconstructed network, and for each combination of region *i* and region *j* where fiber streamlines touched both regions a connection (i.e., network edge) was included in cell *CIJ(i,j)* in the connectivity matrix. Five different types of strength of a connection were computed and included as edge strength: (1) the number of reconstructed streamlines (NOS) between region *i* and *j*, (2) the average FA of the voxels traversed by the reconstructed streamlines, (3) the average MD of the reconstructed streamlines, (4) the average length of the reconstructed streamlines and (5) streamline density computed as the number of reconstructed streamlines corrected for the average volume of region *i* and region *j* (18, 19).

### Outliers

A total of 15,947 connectome maps were analyzed across the participating groups. Of the datasets that could be shared, 197 were detected as outliers (and were subsequently removed from the dataset). Outliers were detected automatically by testing per dataset and for each connectome map their average connection strength and their distance to the group average prevalence map. The average connection strength of a connectome map was calculated for each of the five connection weights as the mean of the strengths over all existing (nonzero) connections. To measure the presence of odd connections or absence of common connections in a connectome map, we constructed a group prevalence matrix for each dataset, counting per node pair how many times an edge was observed across the group of subjects in the dataset. For each connectome map the total prevalence of all observed connections and the total prevalence of all non-observed connections was computed. Outliers were identified as connectome maps that displayed on any of the 7 measures (5 weight and 2 prevalence measures) a score below Q1 – 2 × IQR or above Q3 + 2 × IQR, with Q1 and Q3 referring to the first and third quartile, respectively and IQR the interquartile range IQR = Q3 – Q1. This resulted in the detection of 189 outliers in total, which were excluded from the dataset. One complete dataset (set_634413, n = 584) showed across all included individual sets an average lower FA / higher MD as compared to the other datasets and this set was excluded from the age curves shown in Figure 1. Due to the high overall sample size, including or excluding this dataset did not change the shape of the final plot.

### Cross-Site Comparison

Datasets across sites were compared by computing for each site a group average connectome map (group threshold 60%) and comparing the group average connectivity matrices across each of the sites. Cross-site overlap was computed as the percentage of overlap of the binary matrices and as the correlation between the non-zero elements of the FA group-average matrices.

### Comparison to HCP

To test the validity of the 10K dataset, we compared the group average matrix of the 10K set to the group average matrix of data from the Human Connectome Project (HCP) (3). First, for the 10K dataset, a group average FA matrix was computed, by including those edges that were observed in at least 33% of the group (i.e., a group threshold of 33%, >2700 subjects showing a particular network edge). Average weight values of the included edges were taken as the non-zero mean of those edges across the group of subjects. Second, a similar group average FA matrix was derived from previously analyzed HCP data (22) (*n* = 487 datasets). In brief, HCP analysis included the following steps [see (22) for more detailed information on the HCP data analysis]. For each of the HCP DWI datasets a connectome was reconstructed based on the minimally pre-processed data of HCP. Given the high quality of the HCP data, analysis here included reconstruction of multiple diffusion directions, allowing for the reconstruction of more complex fiber configurations (e.g., crossing fibers) (22). Similarly as for the 10K data, across the total set of 487 datasets, an average FA group matrix was computed, including those network edges that were observed in at least 33% of the total population (i.e., >160 datasets) and taking the non-zero mean of FA values across the group of subjects. Comparison between the 10K set and the HCP dataset was computed by means of (1) counting the number of existing connections and non-existing connections in the 10K dataset as observed in the HCP dataset and (2) by correlating the FA weights of the set of edges as observed in both datasets.

## Ethics Statement

This study was carried out in accordance with the recommendations of the ethical committee boards of each independent institute. The protocol was approved by the ethical committee boards of each independent institute. Informed consent of all participants was acquired by the independent research groups.

## Author Contributions

All authors contributed to the acquisition and/or analysis of the MRI data. MvdH wrote the first draft of the manuscript. All authors revised the manuscript and contributed intellectual content.

### Conflict of Interest Statement

The authors declare that the research was conducted in the absence of any commercial or financial relationships that could be construed as a potential conflict of interest.
